# Genetic Diversity of Campylobacter jejuni and Campylobacter coli Isolates from Conventional Broiler Flocks and the Impacts of Sampling Strategy and Laboratory Method

**DOI:** 10.1128/AEM.03693-15

**Published:** 2016-04-04

**Authors:** A. B. Vidal, F. M. Colles, J. D. Rodgers, N. D. McCarthy, R. H. Davies, M. C. J. Maiden, F. A. Clifton-Hadley

**Affiliations:** a Department of Bacteriology, Animal and Plant Health Agency (APHA), Surrey, United Kingdom; b Department of Zoology, University of Oxford, Oxford, United Kingdom; INRS—Institute Armand-Frappier

## Abstract

The genetic diversity of Campylobacter jejuni and Campylobacter coli isolates from commercial broiler farms was examined by multilocus sequence typing (MLST), with an assessment of the impact of the sample type and laboratory method on the genotypes of Campylobacter isolated. A total of 645 C. jejuni and 106 C. coli isolates were obtained from 32 flocks and 17 farms, with 47 sequence types (STs) identified. The Campylobacter jejuni isolates obtained by different sampling approaches and laboratory methods were very similar, with the same STs identified at similar frequencies, and had no major effect on the genetic profile of Campylobacter population in broiler flocks at the farm level. For C. coli, the results were more equivocal. While some STs were widely distributed within and among farms and flocks, analysis of molecular variance (AMOVA) revealed a high degree of genetic diversity among farms for C. jejuni, where farm effects accounted for 70.5% of variance, and among flocks from the same farm (9.9% of variance for C. jejuni and 64.1% for C. coli). These results show the complexity of the population structure of Campylobacter in broiler production and that commercial broiler farms provide an ecological niche for a wide diversity of genotypes. The genetic diversity of C. jejuni isolates among broiler farms should be taken into account when designing studies to understand Campylobacter populations in broiler production and the impact of interventions. We provide evidence that supports synthesis of studies on C. jejuni populations even when laboratory and sampling methods are not identical.

## INTRODUCTION

Campylobacteriosis is the most commonly reported foodborne bacterial gastrointestinal disease in most developed countries. It is estimated that there are nine million cases of human campylobacteriosis per year in the European Union, resulting in a major economic and disease burden to society (1). Campylobacter jejuni accounts for approximately 90% of human cases, followed by C. coli, which accounts for most of the remainder (2–4). Understanding the relative levels of importance of different infection sources for human disease and identification of transmission pathways are prerequisites for the design and implementation of effective control measures.

Broiler chickens are frequently colonized in the ceca by large numbers of Campylobacter, predominantly C. jejuni and C. coli. In the United Kingdom, the prevalence of Campylobacter-colonized broiler flocks is high, both in cecal samples at time of slaughter (79.2%) (5) and in carcasses after chilling (87.3%) (6). Colonized broilers entering the slaughterhouse are likely to be the main source of carcass contamination during slaughter and an important reservoir for human infection (7). This motivates studies to understand the population structure and farm dynamics of the transmission that produces the colonization patterns entering the slaughter process.

The application of sequence-based typing schemes, such as multilocus sequence typing (MLST), to classify and characterize Campylobacter isolates has resulted in major advances in the understanding of Campylobacter ecology and epidemiology (8). MLST generates data in the form of nucleotide sequence or allelic profiles that are electronically portable and comparable and can be shared via publically accessible online databases (9). These investigations have predominantly focused on the association between genotypes and particular ecological niches or host species with the aim of quantifying the relative contributions of these sources to human disease (10–15). There is increasing evidence that a number of clonal complexes (CCs) associated with human campylobacteriosis are widely disseminated and dominant throughout poultry production (16). However, despite evidence of host association, the relative frequencies of MLST types isolated from broiler flocks differ between countries and over time on individual farms (16–18).

A wide genetic diversity of Campylobacter populations in poultry sources has been reported in different studies performed with a variety of genotyping methods (19), including MLST (20). The Campylobacter populations that infect broiler flocks can be complex, containing multiple genotypes, and flocks may be colonized by a succession of different genotypes over time (17, 21–25). Understanding the genetic diversity of Campylobacter populations in broiler flocks is an essential component of understanding the routes of transmission and the potential for human disease reduction.

Molecular epidemiological studies and source attribution models are generally based on molecular comparisons of isolates from different points along the food chain, with data from different studies often combined to produce data sets describing populations of Campylobacter in different species (12). Similarly, an understanding of on-farm population structures would be supported by the joint analysis of data from different studies. These isolates may arise from different sample types and culture methods, and the representativeness of the sampled strain population is a major issue. Different sampling approaches and laboratory methods are used for detection of Campylobacter (26, 27), and these may influence the apparent diversity of the Campylobacter population studied due to selective isolation of some strains over others from the total population (27, 28). In order to synthesize or to jointly analyze data from different studies, the impact of sampling and laboratory methods on the detection of Campylobacter and inferred population structures needs to be quantified. The present study used MLST to assess the impact of sampling and laboratory methodologies (direct culture versus enrichment) on the Campylobacter populations recovered from broiler flocks. The study also investigated the genetic diversity of C. jejuni and C. coli populations between and within commercial broiler farms before final depopulation.

## MATERIALS AND METHODS

### Sampling and microbiology.

Isolates were obtained from a cross-sectional study of 40 conventional broiler flocks from 17 farms at the end of the rearing period. Details of sample collection and bacteriological culture methods for Campylobacter detection and identification have been more fully described elsewhere (27). Briefly, two flocks or houses per farm were sampled on the same day between June and November. From each flock, 16 samples were collected, including boot swabs moistened in buffered peptone water (BPW) (*n* = 3), Cary-Blair medium (CB) (*n* = 3), maximum recovery diluent (MRD) (*n* = 3), and Exeter broth (EX) (*n* = 3); a pooled cecal sample (*n* = 1); and pooled fecal dropping samples (*n* = 3). Bacteriological culture of boot swabs and cecal samples was performed by direct plating of all samples on modified charcoal cefoperazone desoxycholate agar (mCCDA; Oxoid, Basingstoke, United Kingdom) according to ISO 10272-1:2006 for the detection of Campylobacter spp. In addition, all samples were tested by culture on mCCDA after enrichment in Exeter broth. For logistical reasons, only one colony was selected from each of the suspected Campylobacter-positive samples and subjected to confirmation, species identification, and further characterization by MLST. We assumed that, on average, by systematically typing only one colony per sample from the end of the streak, we would detect the predominant isolates at the relative frequencies at which they occurred in all samples. Confirmation and species identification were carried out using a multiplex PCR based on the detection of partial sequences of two genes that allow the simultaneous identification of C. jejuni (*mapA*) and C. coli (*ceuE*) (29). The spread and undefined growth that Campylobacter cells exhibit on mCCDA plates make it difficult sometimes to select isolated colonies for species identification; therefore, some samples were identified as coinfected. Cultures identified by PCR as “mixed” (and therefore containing C. jejuni and C. coli) were excluded from further characterization by MLST, as we would have had to select one of the two species, thus preventing us from selecting the predominant species in that isolate. In total, 751 Campylobacter isolates (645 C. jejuni and 106 C. coli) from 32 flocks and 17 farms, recovered from different sample types, were characterized by MLST. The number of C. jejuni and C. coli isolates characterized per flock and sample type is shown in Table S1 in the supplemental material.

### DNA preparation.

Isolates which had been stored at −80°C in a cryopreservative medium were revived on blood agar (CM0055; Oxoid) (7% sheep blood and 100 mg of cycloheximide per liter) and incubated at 41.5 ± 1°C in a microaerobic atmosphere (84% N_2_–10% CO_2_–6% O_2_) generated in a gas-charged incubator (Heraeus; Thermo, Basingstoke, United Kingdom). Chromosomal DNA was extracted by boiling a cell suspension in phosphate-buffered saline (PBS) (0.1 M, pH 7.2) for 10 min on a heat block. The suspension was centrifuged at 13,200 rpm for 5 min, and the supernatant was retained frozen until MLST analysis.

### MLST.

MLST was performed as described previously (30). Fragments of seven housekeeping genes (aspartase A [*aspA*], glutamine synthetase [*glnA*], citrate synthase [*gltA*], serine hydroxymethyl transferase [*glyA*], phosphoglucomutase [*pgm*], transketolase [*tkt*], and ATP synthase alpha subunit [*uncA*]) were amplified by PCR, and the nucleotide sequence of the amplicons was determined using published oligonucleotide primers and reaction conditions (30, 31). Nucleotide sequence extension reaction products were separated and detected with an ABI 3730 automated DNA analyzer using a BigDye Terminator v3.1 cycle sequencing kit (ThermoFisher Scientific; catalog no. 4337457). The consensus sequence was queried against the Campylobacter database to give an allele number. The combination of alleles for the seven housekeeping genes gave the ST. STs are assigned to genetically related CCs based on sharing four or more alleles with the defined central genotype. Multilocus sequence typing alleles, STs, and CCs were assigned using the Campylobacter PubMLST database (http://pubmlst.org/campylobacter) with sequences submitted for allele designation as appropriate.

### Statistical analysis. (i) Analysis of genetic differentiation.

Nucleotide-based analyses of gene flow and genetic differentiation were performed using the pairwise Fisher statistic (F_*ST*_). A value of 0 indicates that two populations are indistinguishable, while a value of 1 indicates maximum genetic differentiation between two populations. The resulting pairwise F_*ST*_ values were exported as genetic distances and visualized as neighbor-joining trees in MEGA5 (32). Additional analysis of genetic subdivision and analysis of molecular variance (AMOVA) were performed. AMOVA analyses the variance in frequencies of concatenated MLST alleles within and between populations or groups by dividing the value corresponding to the total variance into different covariance components corresponding to different levels of a hierarchical population structure (between flocks, between flocks within the same farm, and between farms). The significance of the variance components was tested using nonparametric permutation (1,023 permutations) to obtain a null distribution of the given hierarchy. F_*ST*_ analysis and AMOVA were performed using Arlequin software version 3.5.1.2 (33, 34).

### (ii) Analysis of genetic diversity.

To test whether particular samples and laboratory methods were more or less likely to harbor particular STs, the data were collapsed into 2-by-2 tables to compare the frequency of each ST from each sample type with the total frequency observed in the other samples. The chi-square statistic was used to test the distribution of STs recovered from different sample types and laboratory methods. Where the value corresponding to an observation was less than 5, Fisher's exact test statistic was used. Fisher's exact tests and chi-square analysis were performed using Stata version 12 (StataCorp LP, TX, USA).

A modified version of Simpson's diversity index (1-D) and bootstrap 95% confidence intervals were calculated using StatsDirect (StatsDirect Ltd., Cheshire, United Kingdom) to compare the diversities of STs within the Campylobacter populations isolated from different samples and laboratory methods. This index takes into account the number of STs present (the “richness”) as well as the abundance (the “evenness”) for each ST. The Simpson's diversity index represents the probability that two individuals randomly selected from a sample belong to different species. The value ranges between 0 and 1; the greater the value, the greater the sample diversity (35, 36).

Rarefaction analysis was performed by using the frequency of STs to investigate the relative levels of diversity or species richness among different sample types and laboratory methods. The rarefaction analysis was carried out using PAST (37) with a rarefaction function. In rarefaction analysis, the horizontal axis of the plot represents the number of samples used for analysis and the vertical axis represents the diversity or the number of STs identified in the specified number of samples.

## RESULTS

### Diversity of sequence types.

**FIG 1:**
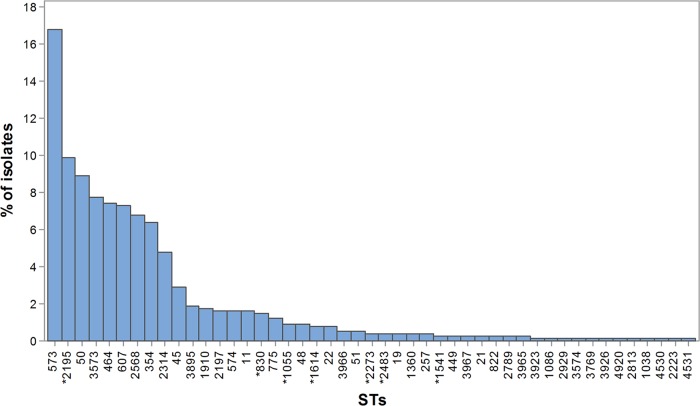
Frequency distribution of C. jejuni (*n* = 645) and C. coli (*) (*n* = 106) STs from 32 broiler flocks.

A total of 47 STs (40 C. jejuni and 7 C. coli) were identified among the 751 isolates and assigned to 15 CCs (14 C. jejuni and 1 C. coli). Six STs (five C. jejuni and one C. coli) accounting for 26.2% of the total isolates (18.4% of C. jejuni and 69.8% of C. coli isolates) did not belong to a known clonal complex at the time of the analysis and therefore do not have an assigned clonal complex. A total of 21 of the STs (17 C. jejuni and 4 C. coli) were represented by 5 or more isolates. The most common ST was ST-573 (126 of 751; 16.8%), followed by ST-2195 (74 of 751; 9.9%) and ST-50 (67 of 751; 8.9%). Eight STs (ST-573, ST-2195, ST-50, ST-3573, ST-464, ST-607, ST-2568, and ST-354) accounted for more than 70% of the isolates (Fig. 1). The number of STs identified from each represented CC ranged from 1 (ST-48, ST-52, ST-574, and ST-607 complexes) to 12 (ST-21 complex). The CCs were represented by between 4 and 128 isolates (see Table S2 to S4 in the supplemental material).

### Campylobacter genotypes from different sample types.

**FIG 2:**
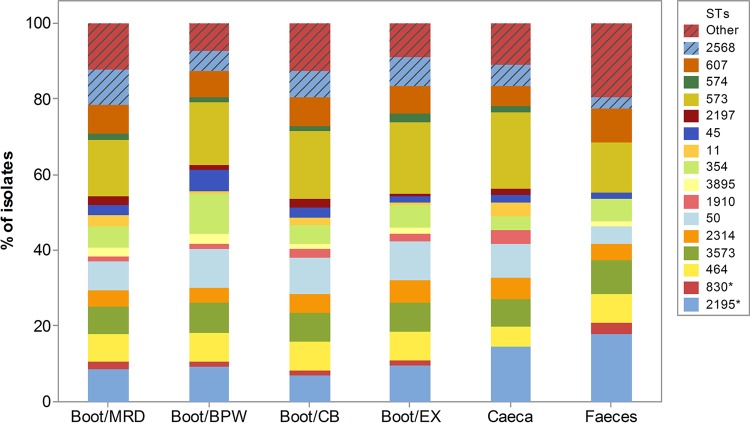
Bar chart showing the frequency distribution of C. jejuni (*n* = 645) and C. coli (*) (*n* = 106) STs between sample types. STs identified in 10 or fewer isolates were grouped into the category “Other.”

A total of 282 chi-square tests were carried out to compare the frequency distributions of STs among different sample types, and only three STs showed a statistically significant difference in the distribution between sample types. ST-354 and ST-45 were more frequently recovered from boot swabs moistened in BPW (*P* = 0.0218 and *P* = 0.015, respectively), and the frequency of ST-2195 was significantly higher in feces (*P* = 0.036) than in all the other sample types. Interestingly, feces was the only sample type that did not yield ST-574, ST-1910, ST2197, ST-11, and ST-48 (Fig. 2; see also Table S2 in the supplemental material).

Simpson's index of diversity for C. jejuni isolates was largely unaffected by the sample method, ranging among the eight approaches used from 0.91 for boot swabs transported in EX, CB, and BPW to 0.92 for boot swabs transported in MRD and feces (see Table 2). The genetic diversity was lower for C. coli isolates for all the sample types and ranged from 0.39 (confidence interval [CI], 0.13 to 0.67) for boot swabs transported in MRD to 0.69 (CI, 0.48 to 0.90) for boot swabs moistened in CB, with the confidence intervals of one method overlapping the point estimate of the other in all comparisons.

F_*ST*_, the genetic fixation index, was used to quantify the extent of genetic differentiation among populations. The index ranges from 0 to 1; a value of 1 indicates that the populations are completely different or separate (33). No genetic differentiation was observed between C. jejuni isolates from different sample types with F_*ST*_ values not statistically different from 0 (Table 1). In comparisons of C. coli isolates from different sample types, F_*ST*_ values ranged from 0 to 0.046, although none of these values were statistically significant (Table 1).

**TABLE 1:** Population pairwise F*_ST_* for C. jejuni and C. coli isolates from different sample types^*a*^

Species	Sample type^*b*^	Population pairwise F*_ST_*					
		Boot/MRD	Boot/EX	Boot/BPW	Boot/CB	Cecum	Feces
*C*. jejuni	Boot/MRD	0	0.956	0.708	0.996	0.928	0.846
	Boot/EX	0	0	0.83	0.999	0.994	0.812
	Boot/BPW	0	0	0	0.871	0.776	0.841
	Boot/CB	0	0	0	0	0.998	0.913
	Cecum	0	0	0	0	0	0.881
	Feces	0	0	0	0	0	0
*C*. coli	Boot/MRD	0	0.553	0.645	0.594	0.277	0.919
	Boot/EX	0	0	0.999	0.449	0.699	0.919
	Boot/BPW	0	0	0	0.25	0.651	0.801
	Boot/CB	0	0	0.014	0	0.206	0.847
	Cecum	0.025	0	0	0.046	0	0.514
	Feces	0	0	0	0	0	0

a F*_ST_* values and *P* values are shown in the lower and upper half of the diagonal matrix, respectively.

b Boot/MRD, boot swabs moistened in MRD; Boot/Ex, boot swabs moistened in Exeter broth; Boot/BPW, boot swabs moistened in BPW; Boot/CB, boot swabs moistened in Cary-Blair medium.

Rarefaction curves showing the diversity of STs as a function of the number of isolates for each sample were generated (see Fig. S1 in the supplemental material). A slope of zero in the rarefaction curves indicated that the maximum genetic diversity had been reached and that it was unlikely that more genetic diversity would be identified if more samples were analyzed. The rarefaction analysis did not show any significant differences in diversity between sample types, although a trend toward greater diversity for C. jejuni genotypes in cecal and fecal samples than in boot swab samples was observed. For C. coli, greater genotype richness was observed for boot swabs transported in CB than for the other samples, although these differences were not statistically significant.

### Campylobacter genotypes from different laboratory methods.

Of the total of 47 STs isolated in this study, 8 were recovered by direct culture alone, 20 were recovered after enrichment only, and 19 were recovered by both methods. Multiple comparisons of the frequency distributions of STs between direct culture and enrichment revealed that only a few STs were statistically more frequently isolated using one method than using the other. ST-22, ST-48, and ST-830 were more frequently isolated from enrichment (six, seven, and eight isolates, respectively) than by direct culture (none), and these differences were statistically significant (*P* = 0.03, *P* = 0.017, and *P* = 0.009, respectively). C. jejuni ST-775 was the only type significantly more frequently identified after direct culture than after enrichment (*P* = 0.001) (see Table S3 in the supplemental material).

The Simpson's index of diversity for C. jejuni was 0.90 (CI, 0.88 to 0.92) for isolates recovered by direct culture and 0.92 (CI, 0.91 to 0.93) for those obtained after enrichment (Table 2). For C. coli isolates, greater genetic diversity was observed after enrichment (0.61; CI, 0.49 to 0.63) than by direct culture (0.31; CI, 0.14 to 0.48), although the confidence intervals of one overlapping the point estimate again indicated that these differences were not statistically significant (Table 2). A small but significant degree of differentiation between direct culture and enrichment was observed for C. coli isolates (F_*ST*_ = 0.08; *P* = 0.0059). No significant difference was observed between direct culture and enrichment for C. jejuni isolates (F_*ST*_ = 0.00089; *P* = 0.2423). Rarefaction analysis showed a greater diversity of C. jejuni and C. coli genotypes after enrichment than after direct culture (see Fig. S2 in the supplemental material). However, the confidence intervals of the rarefaction curves overlapped in both cases (data not shown), indicating that these differences should be interpreted with care.

**TABLE 2:** Diversity indices of C. jejuni and C. coli populations from different sample types and laboratory methods^*a*^

Sample type	C. jejuni			C. coli		
	1-D	95% CI		1-D	95% CI	
		Lower	Upper		Lower	Upper
Boot/MRD	0.92	0.91	0.94	0.53	0.32	0.76
Boot/EX	0.91	0.88	0.93	0.44	0.18	0.70
Boot/CB	0.91	0.89	0.94	0.69	0.48	0.90
Boot/BPW	0.91	0.89	0.93	0.39	0.13	0.67
Feces	0.92	0.89	0.96	0.56	0.30	0.81
Cecum	0.92	0.87	0.97	ND	ND	ND
DC	0.90	0.88	0.92	0.31	0.14	0.48
EN	0.92	0.91	0.93	0.61	0.49	0.73

a Boot/MRD, boot swabs moistened in MRD; Boot/Ex, boot swabs moistened in Exeter broth; Boot/BPW, boot swabs moistened in BPW; Boot/CB, boot swabs moistened in Cary-Blair medium; DC, direct culture; EN, enrichment; ND, not determined (the number of MLST types was too low to estimate 1-D value and 95% CI).

### Campylobacter genotypes among and within farms.

The number of CCs within a flock and within a farm ranged from 1 to 6 and from 1 to 7, with averages of 1.9 and 2.6, respectively. Some of the CC and STs were widely distributed within and between flocks and farms. CC573 was found in five farms and nine flocks, CC353 and CC354 were each found in five farms and six flocks, and CC21 was found in four farms and six flocks. The number of STs within a flock and within a farm ranged from 1 to 8, with averages of 4.4 STs per farm and 3.1 STs per flock. Although some STs were widely distributed within and between farms and flocks (ST-573 was found in 9 flocks from five farms, and ST-2195 was found in 11 flocks from eight farms), only 10 of 47 (21.3%) STs were found on two or more farms. Other STs (ST-464, ST-3573, and ST-607) were found in only one particular farm, where they predominated (see Table S4 in the supplemental material).

**FIG 3:**
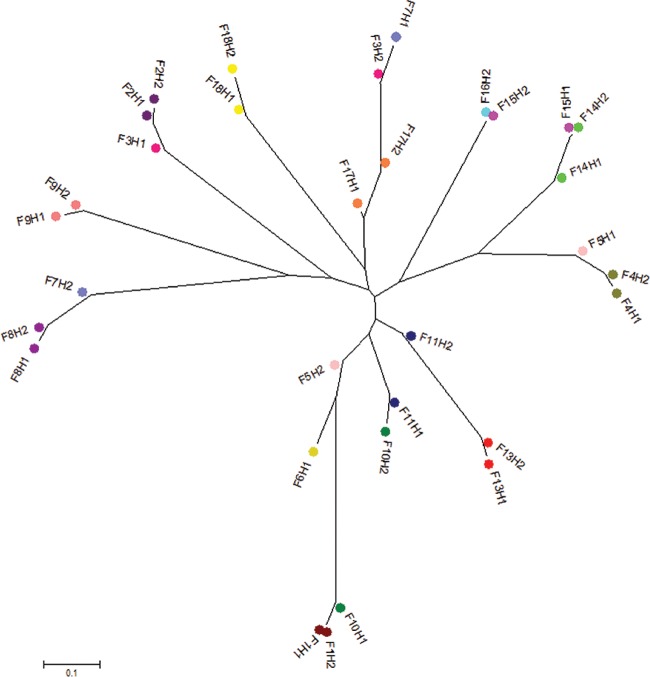
Unrooted neighbor-joining tree displaying the pairwise genetic distances (F_*ST*_ values) between C. jejuni populations from different flocks (H) from different farms (F). The same color represents isolates from flocks from the same farm. The F_*ST*_ values were calculated from nucleotide polymorphisms in the concatenated sequences from seven loci in the 645 C. jejuni isolates. All differences between flocks were significant at a *P* value of <0.05.

**FIG 4:**
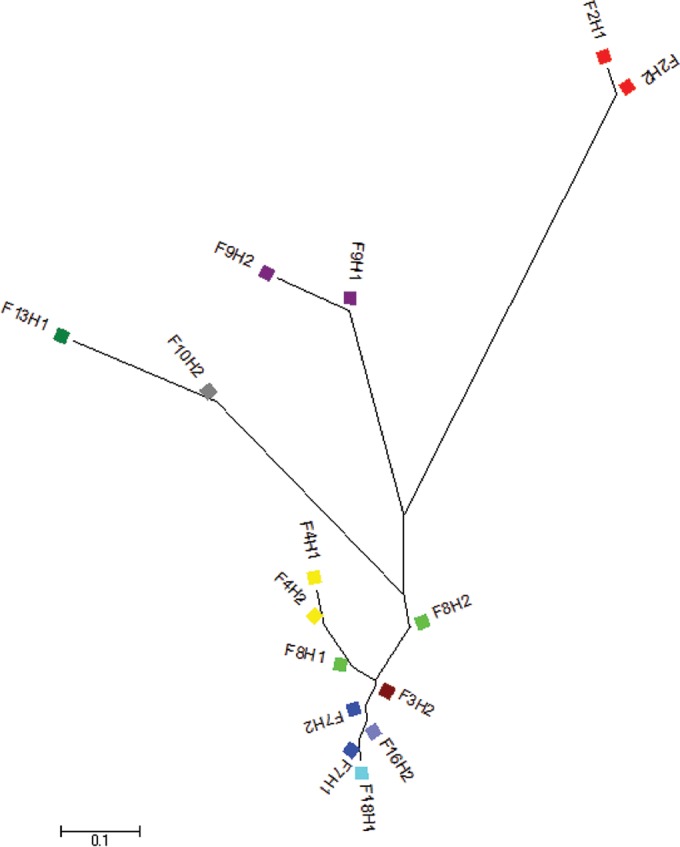
Unrooted neighbor-joining tree displaying the pairwise genetic distances (F_*ST*_ values) between C. coli populations from different flocks from different farms. The same color represents isolates from flocks from the same farm. The F_*ST*_ values were calculated from nucleotide polymorphisms in the concatenated sequences from seven loci in the 106 C. coli isolates. All differences between flocks were significant at a *P* value of <0.05.

The AMOVA showed significant differentiation of C. jejuni isolates for the different levels of the population structure, with strong evidence of between-farm variability, which accounted for 70.5% of the total variance. There was also strong evidence of between-flock diversity within farms and of diversity within flocks, although this accounted for much smaller proportions of the total variance (9.9% and 19.6%, respectively) (Table 3). No effect of farm differences was seen for C. coli, and the variance was best explained by variation between flocks within the same farm (64.1%) and within flocks (25.1%) (Table 3). Figures 3 and 4 represent the F_*ST*_ population differentiation as a NeighborNet tree, whereby the distance between nodes represents the degree of differentiation at the population level between flocks from different farms.

**TABLE 3:** AMOVA model results showing the hierarchical partitioning of the variance in STs of C. jejuni and C. coli isolates originating from 17 farms and 32 flocks^*a*^

Species	Source of variation	df	Sum of squares	Variance components	% variation	*P* value
*C*. jejuni (*n* = 645)	Between farms	15	9,795.9	15.02	70.52	<0.0001
	Between flocks within farms	15	677.3	2.11	9.92	<0.0001
	Within flocks	616	2,564.7	4.16	19.56	<0.0001
	Total	646	13,038	21.29		
*C*. coli (*n* = 106)	Between farms	8	14.61	0.03	10.84	NS
	Between flocks within farms	5	5.15	0.18	64.05	<0.0001
	Within flocks	91	6.28	0.07	25.11	<0.0001
	Total	104	26.04	0.27		

a AMOVA, analysis of molecular variance; NS, not significant.

## DISCUSSION

This study assessed the effect of the sample type and laboratory method on the molecular types recovered from conventional broiler flocks and examined the genetic diversity of C. jejuni and C. coli at the farm and flock levels.

The Campylobacter populations identified by a high number of different sampling approaches (boot swabs, cecal content, and feces) were very similar, with far smaller differences among sample types than among different farms. Only three STs (ST-354, ST-45, and ST-2195) were statistically more frequently identified in one sample type than in the others; however, due to the high number of multiple comparisons, these significant differences may have been due to chance. For C. jejuni isolates, similar and high degrees of genetic diversity were observed in isolates from broiler flocks by the use of different sampling strategies and no evidence of genetic differentiation among sample types was detected. Similarly, the rarefaction analysis showed marginal differences between the relative diversities of STs obtained by each sample type when equal numbers of samples were used. Lower levels of genetic diversity and higher levels of differentiation were observed between C. coli populations from different sample types, but these differences were not statistically significant. For C. coli, our results are therefore consistent with anything from a minimal to a substantial impact of sampling. Overall, these findings indicate that the use of boot swab, cecal, or fecal samples for isolation of C. jejuni at the farm level had no discernible effect on the genetic profile of the flocks.

The genotypic profiles of the Campylobacter population from direct culture and enrichment were very similar, with the two methods identifying the main types and at similar frequencies. It has been suggested that the isolation protocols used with enrichment may bias the recovery toward C. jejuni genotypes with increased laboratory fitness, thus limiting the understanding of population genetics and genetic diversity of Campylobacter strains circulating in environmental and animal reservoirs (28). In the present study, the STs identified by only one culture method were usually detected in very few (fewer than 5) samples for all but four of the STs (ST-830, ST-22, ST-48, and ST-775) that were significantly more commonly recovered after enrichment. Most of these STs are known to occur in human disease and have been identified in other studies, with ST-830 more typically isolated from pigs and ST-22 and ST-48 more typically isolated from cattle and sheep (pubMLST isolate database). This may support the hypothesis of a better performance of the enrichment method for the recovery of genotypes that are not commonly found in chickens and that are therefore expected to be present at low concentration in the samples.

No significant differentiation of genotypes obtained by different laboratory methods was observed for C. jejuni. However, a small but significant degree of differentiation between direct culture and enrichment was observed for C. coli isolates, probably due to the increased isolation rate of ST-830 after enrichment, as discussed above. An increase in the genetic diversity after enrichment was observed for C. jejuni and C. coli, although the 95% confidence intervals overlapped, indicating a lack of statistical significance in both cases. Overall, the findings suggest that enrichment may be more sensitive at picking up strains that are present at low concentration in the samples, but the present study did not demonstrate a substantial bias in the nature of the strains identified by these two methods, so the impact on the inferred population structure should be small, in particular, for C. jejuni.

Williams et al. (28) described greater genetic diversity in isolates from farm environmental samples (swabs, feed, and water) when the samples were enriched in Exeter broth for both C. jejuni and C. coli isolates. In contrast, a higher level of genotypic richness has been reported by direct plating of neck skin, cecum, and meat samples than by enrichment in Preston and Bolton broths (38). The available methods are not necessarily optimal for the recovery of campylobacters from the whole range of sample types (39), and optimal culture methods often appear to be sample type specific and their performance dependent largely on the numbers of the bacteria or genotype present, their viability, and the presence of other competing organisms in the sample.

A few studies have compared the genetic diversities of Campylobacter populations from different sampling sites and sample matrices across the broiler food chain, but, to our knowledge, there are no published studies comparing different sample types from broiler flocks at the farm level. A recent study comparing cecal, neck skin, and meat samples found a greater level of genetic diversity in isolates from neck skin and cecal samples than in those from meat samples (38). Others have found that the diversity increases from the farm to slaughter, suggesting that the full diversity of Campylobacter genotypes found at slaughter may not be captured by on-farm sampling, although cross-contamination during the slaughter process is also likely to be responsible for the increased diversity (14). This could mean that characterization of isolates from on-farm monitoring may help facilitate studies of dissemination pathways.

Poultry samples have been found to be contaminated with more than one ST (11, 40); therefore, the number of isolates chosen per sample may also influence the population diversity observed. In the present study, only one colony was picked per positive sample and it was therefore not possible to assess the heterogeneity of the population within each of the samples and its impact on the overall genetic diversity of the Campylobacter population in broiler flocks.

Despite the great genetic diversity observed here, the presence of a limited number of predominant types shared by multiple flocks was also observed. Common poultry management practices may favor the recirculation of these strains among and within farms. The use of common vehicles and personnel across different farms, particularly in high-density poultry areas and within integrated companies, may have contributed to the transmission of particular Campylobacter strains. The predominance of a particular genotype within flocks and farms may also suggest the presence of a more stable, adapted, and successful flock colonizer.

The analysis of molecular variance showed that the genetic variation of C. jejuni isolates resided mainly among farms, with less variation observed within flocks and between flocks within the same farm. Other studies have identified a spatial relationship among genotypes, with isolates being more similar within rather than between cattle and sheep farms (41–44). However, to our knowledge there is no evidence of these types of studies being carried out in broiler farms. The network between abattoirs and farms, particularly within the same company, as was the case in the present study, together with the environmental characteristics of the area may explain the dissemination and perpetuation of certain strains in the particular geographic area where each of the study farms was located. Different strains may respond differently to certain environmental conditions and management practices, which, combined with different survival and invasion characteristics, may explain their particular distributions among farms. Interestingly, for C. coli isolates, the majority of genetic variation occurred among flocks within the same farm, with the within-flock diversity accounting for a much smaller proportion of the variation. These findings are in line with other work which suggested that C. coli may form a more stable population within a broiler flock than C. jejuni (45).

The dynamics of strain diversity within flocks are complex and may reflect variation in phenotypic properties such as infectivity, virulence, and stress response, which could determine survival time, persistence, or different susceptibility properties. Several groups have also demonstrated that the genotype can affect colonization of the gastrointestinal tract of poultry (46–49) and that passage through poultry can affect both the genotype and the colonization of poultry (50–53).

Interrogation of the Campylobacter MLST database revealed that most of the STs and CCs identified in this study have been previously described among isolates from poultry and humans, reinforcing the hypothesis of the importance of broiler flocks as a reservoir for human infection. Interestingly, other chicken-associated lineages that have been previously reported frequently were not identified in the farms in the fairly large current study. This shows the need for very wide on-farm sampling to fully index the Campylobacter population affecting the broiler industry at the farm level.

This report shows that the use of different sampling strategies and laboratory methods for isolation of Campylobacter at the farm level has no statistically supported effect on the genetic profile of C. jejuni population in broiler flocks. The report also shows that commercial broiler farms provide an ecological niche for a wide diversity of genotypes and illustrates the complexity of the population structure of these organisms in the broiler production. The higher level of genetic diversity between broiler farms should be taken into account in designing sampling strategies to understand the population structure of Campylobacter in the broiler production. Further investigations will be needed to better understand the factors responsible for the genetic diversity of C. jejuni and C. coli between and within broiler farms and flocks and the impact of control interventions altering Campylobacter populations as well as the overall quantitative impact of any interventions.

## Supplementary Material

Supplemental material
