# Supplementary material for: Genetic Diversity of Campylobacter jejuni and Campylobacter coli Isolates from Conventional Broiler Flocks and the Impacts of Sampling Strategy and Laboratory Method
Source: Appl Environ Microbiol. 2016 Apr 4;82(8):2347–55. doi: 10.1128/AEM.03693-15 (PMC4959481; doi:10.1128/AEM.03693-15)
Supplement: Supplemental material [file supp_82_8_2347__index.html]

Genetic Diversity of Campylobacter jejuni and Campylobacter coli Isolates from Conventional Broiler Flocks and the Impacts of Sampling Strategy and Laboratory Method — Supplemental material 

# Genetic Diversity of Campylobacter jejuni and Campylobacter coli Isolates from Conventional Broiler Flocks and the Impacts of Sampling Strategy and Laboratory Method

## Supplemental material

- Supplemental file 1 -

  *Campylobacter* isolates from 32 broiler flocks characterized by MLST and their distribution by sample type (Table S1), distributions of STs and resolution into clonal complexes (Tables S2 to S4), and rarefaction curves (Fig. S1 and S2).

  PDF, 306K
