# Supplementary material for: Genetic Diversity of Campylobacter jejuni and Campylobacter coli Isolates from Conventional Broiler Flocks and the Impacts of Sampling Strategy and Laboratory Method
Source: Appl Environ Microbiol. 2016 Apr 4;82(8):2347–55. doi: 10.1128/AEM.03693-15 (PMC4959481; doi:10.1128/AEM.03693-15)
Supplement: Supplemental material [file AEM.03693-15_zam999117067so1.pdf]

Genetic diversity of *Campylobacter jejuni* and *Campylobacter coli* isolates from conventional broiler flocks and the impact of sampling strategy and laboratory method

Supplementary information

Table S1. *Campylobacter* isolates (n=751) from 32 broiler flocks characterised by MLST and their distribution by sample type

Farm/House	Boot/BPW	Boot/CB	Boot/EX	Boot/MRD	Caeca	Char-swab	Total
F1H1	6	6	6	6	2	2	28
F1H2	6	6	6	6	2	3	29
F2H1	2	3	3	4			12
F2H2	2	2	1	2			7
F3H1	6	6	6	6	2	3	29
F3H2	4	3	3	4	2		16
F4H1	5	4	4	4	2	1	20
F4H2	4	4	4	6	2		20
F5H1		1			2	3	6
F4H2	6	6	6	6	2	3	29
F6H1	6	6	6	6			24
F7H1	3	4	4	3	1	1	16
F7H2	4	3	3	6	1	2	19
F8H1	5	5	6	6	2	2	26
F8H2	4	6	4	4	2	3	23
F9H1	6	5	4	5	2	2	24
F9H2	3	4	3	6	2	3	21
F10H1	5	6	6	5	2	3	27
F10H2	4	4	5	6	1	2	22
F11H1	6	6	6	5	2	1	26
F11H2	6	6	6	6	2	2	28
F13H1	2	4	4	3	2	1	16
F13H2	6	6	6	6	2	3	29
F14H1	6	6	6	6	2	3	29
F14H2	6	6	6	6	2	3	29
F15H1	6	5	6	5	2	3	27
F15H2	5	6	6	6	2	3	28
F16H2	6	5	6	4	2	3	26
F17H1	6	6	6	6	2	3	29
F17H2	6	6	6	6	2	3	29
F18H1	5	6	6	6	2	3	28
F18H2	6	6	6	6	2	3	29
Total	153	158	156	162	55	67	751

Table S2. Distribution of STs among *C. jejuni* (n=645) and *C. coli* (n=106) isolates obtained from different sample types and their resolution into clonal complexes

Species	Clonal complex	ST	Sample type						Total
			Boot/BPW	Boot/CB	Boot/EX	Boot/MRD	caeca	Char-swab	
C. coli	Unassigned	2195	14	11	15	14	8	12	74
	ST-828	830	2	2	2	3		2	11
		1055		2	1	1	1	2	7
		1541		2					2
		1614	1	1		3		1	6
		2273		1	1			1	3
		2483	1	1	1				3
C. jejuni	Unassigned	449	1			1			2
		464	12	12	12	12	3	5	56
		3573	12	12	12	12	4	6	58
		3923					1		1
		3967					1	1	2
	ST-1034	2314	6	8	9	7	3	3	36
	ST-21	19			1	2			3
		21	2						2
		50	16	15	16	12	5	3	67
		822				2			2
		1086				1			1
		1360	1	1				1	3
		2789		1		1			2
		2929						1	1
		3574						1	1
		3769						1	1
		3926		1					1
		3966	1	1	1			1	4
	ST-22	22	1	2	1	1		1	6
	ST-257	257		2	1				3
		4920				1			1
	ST-353	1910	2	4	3	2	2		13
		2813					1		1
		3895	4	2	3	4		1	14
	ST-354	354	16	8	9	9	2	4	48
		1038				1			1
		4530			1				1
	ST-443	51	1	1		2			4
	ST-45	11	1	3	1	5	2		12
		45	9	4	3	4	1	1	22
		2197	2	4	1	4	1		12
		2223					1		1
	ST-48	48	1	1	3	1	1		7
	ST-52	775	1	2	3	3			9
	ST-573	573	25	28	29	24	11	9	126
		3965		1				1	2
	ST-574	574	2	2	4	3	1		12
	ST-607	607	11	12	11	12	3	6	55
	ST-661	2568	8	11	12	15	3	2	51
		4531						1	1
Total			153	158	156	162	55	67	751

Table S3. Distribution of STs among *C. jejuni* (n=645) and *C. coli* (n=106) isolates obtained after direct culture and enrichment and their resolution into clonal complexes

Species	Clonal complex	ST	Laboratory methodology		Total
			DC	EN	
C. coli	Unassigned	2195	39	35	74
	ST-828	830		11	11
		1055	2	5	7
		1541	2		2
		1614	2	4	6
		2273	2	1	3
		2483		3	3
C. jejuni	Unassigned	449		2	2
		464	25	31	56
		3573	26	32	58
		3923		1	1
		3967		2	2
	ST-1034	2314	20	16	36
	ST-21	19		3	3
		21	2		2
		50	31	36	67
		822		2	2
		1086	1		1
		1360		3	3
		2789		2	2
		2929		1	1
		3574		1	1
		3769		1	1
		3926		1	1
		3966		4	4
	ST-22	22		6	6
	ST-257	257	3		3
		4920		1	1
	ST-353	1910	7	6	13
		2813	1		1
		3895	7	7	14
	ST-354	354	27	21	48
		1038	1		1
		4530	1		1
	ST-443	51	2	2	4
	ST-45	11	5	7	12
		45	10	12	22
		2197	3	9	12
		2223		1	1
	ST-48	48		7	7
	ST-52	775	9		9
	ST-573	573	66	60	126
		3965		2	2
	ST-574	574	7	5	12
	ST-607	607	25	30	55
	ST-661	2568	19	32	51
		4531		1	1
Total			345	406	751

Table S4. Distribution of *C. jejuni* (n=645) and C. coli (n=106) STs and CCs amongst 17 broiler farms and 32 broiler flocks

Species	CC	ST	Farm/House																																
			F1H1	F1H2	F2H1	F2H2	F3H1	F3H2	F4H1	F4H2	F5H1	F5H2	F6H1	F7H1	F7H2	F8H1	F8H2	F9H1	F9H2	F10H1	F10H2	F11H1	F11H2	F13H1	F13H2	F14H1	F14H2	F15H1	F15H2	F16H2	F17H1	F17H2	F18H1	F18H2	Total
C. coli	Un*	2195						4	5	1				5	3	9	9		8					3						26			1		74
	ST-828	830																			11														11
		1055																7																	7
		1541			2																														2
		1614														4	2																		6
		2273							3																										3
		2483				3																													3
C. jejuni	Un*	449				2																													2
		464	28	28																															56
		3573																													29	29			58
		3923						1																											1
		3967																							2										2
	ST-1034	2314																		25	11														36
	ST-21	19																						1	2										3
		21						2																											2
		50									4	26	20											6	11										67
		822																						2											2
		1086											1																						1
		1360									1	1	1																						3
		2789											1											1											2
		2929										1																							1
		3574										1																							1
		3769									1																								1
		3926																						1											1
		3966																							4										4
	ST-22	22																								6									6
	ST-257	257						3																											3
		4920						1																											1
	ST-353	1910												11	2																				13
		2813		1																															1
		3895					1		12				1																						14
	ST-354	354					17	1		7					9					1						13									48
		1038																		1															1
		4530													1																				1
	ST-443	51													4																				4
	ST-45	11								12																									12
		45					4	2										15	1																22
		2197																	12																12
		2223																1																	1
	ST-48	48																				6												1	7
	ST-52	775					7	2																											9
	ST-573	573														8	10	1				18	28			1	5	27	28						126
		3965																				2													2
	ST-574	574																						2	10										12
	ST-607	607																															27	28	55
	ST-661	2568			10	2										5	2									9	23								51
		4531																									1								1
Total			28	29	12	7	29	16	20	20	6	29	24	16	19	26	23	24	21	27	22	26	28	16	29	29	29	27	28	26	29	29	28	29	751

a) b)

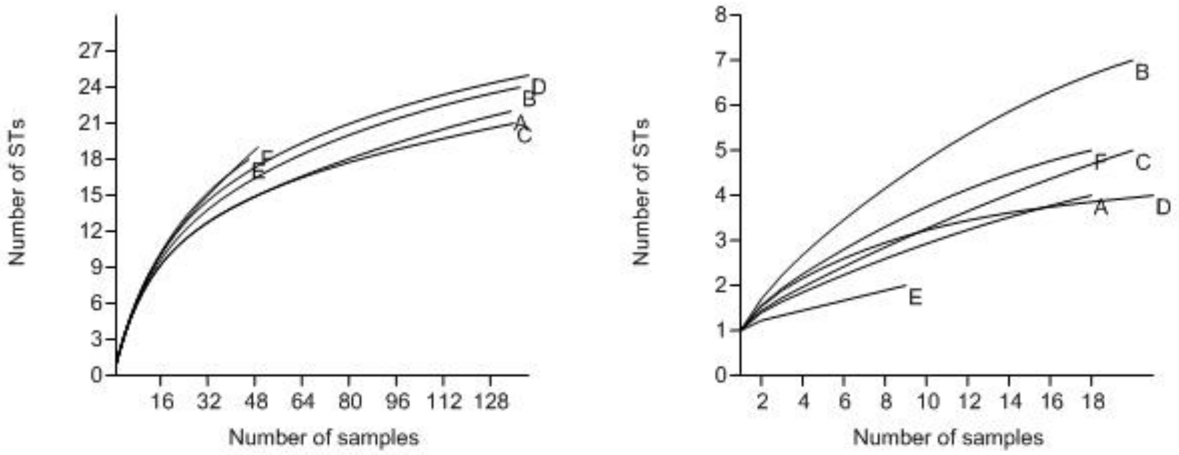

Figure S1. Rarefaction curves of *C. jejuni* (a) and *C. coli* (b) sequence types (STs) isolated from boot/BPW (A), boot/CB (B), boot/EX (C), boot/MRD (D), Caeca (E), faecal (F) samples. The X-axis denotes the number of samples genotyped and the Y-axis denotes the number of different STs present in the given number of isolates.

a) b)

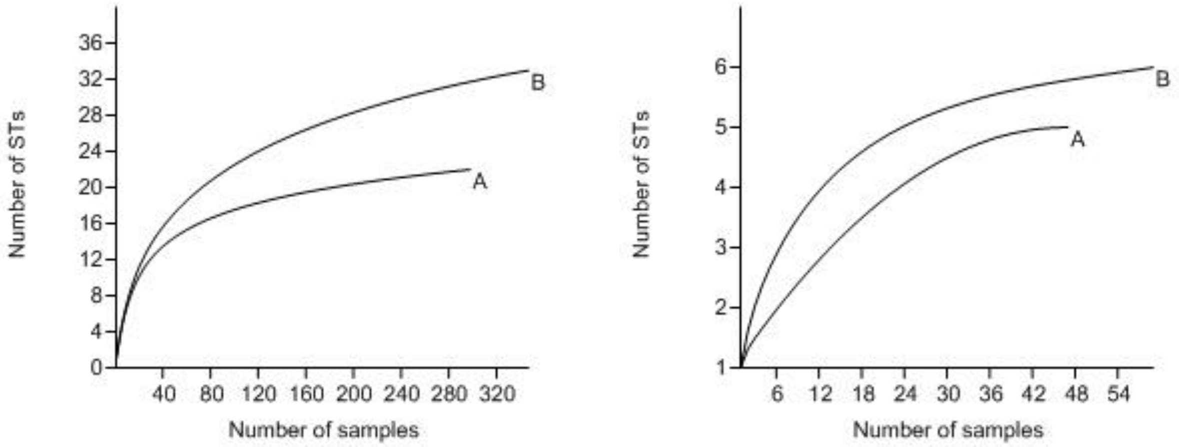

Figure S2. Rarefaction curves of *C. jejuni* (a) and *C. coli* (b) sequence types (STs) isolated from direct culture (A), and after enrichment in Exeter broth (B). The X-axis denotes the number of samples genotyped and the Y-axis denotes the number of different STs present in the given number of isolates.
